# High-temperature superconductivity in space-charge regions of lanthanum cuprate induced by two-dimensional doping

**DOI:** 10.1038/ncomms9586

**Published:** 2015-10-20

**Authors:** F. Baiutti, G. Logvenov, G. Gregori, G. Cristiani, Y. Wang, W. Sigle, P. A. van Aken, J. Maier

**Affiliations:** 1 Max Planck Institute for Solid State Research, Heisenbergstrasse 1, Stuttgart 70569, Germany; 2 Max Planck Institute for Intelligent Systems, Heisenbergstrasse 3, Stuttgart 70569, Germany

## Abstract

The exploitation of interface effects turned out to be a powerful tool for generating exciting material properties. Such properties include magnetism, electronic and ionic transport and even superconductivity. Here, instead of using conventional homogeneous doping to enhance the hole concentration in lanthanum cuprate and achieve superconductivity, we replace single LaO planes with SrO dopant planes using atomic-layer-by-layer molecular beam epitaxy (two-dimensional doping). Electron spectroscopy and microscopy, conductivity measurements and zinc tomography reveal such negatively charged interfaces to induce layer-dependent superconductivity (*T*_c_ up to 35 K) in the space-charge zone at the side of the planes facing the substrate, where the strontium (Sr) profile is abrupt. Owing to the growth conditions, the other side exhibits instead a Sr redistribution resulting in superconductivity due to conventional doping. The present study represents a successful example of two-dimensional doping of superconducting oxide systems and demonstrates its power in this field.

## Abstract (web summary)

Introduction of higher-dimensional structure elements into solids is used to generate unusual materials properties. Here, the authors report how replacing LaO planes with SrO dopants yields space-charge induced superconductivity, showing the potential of two-dimensional doping in this field.

## Graphical abstract

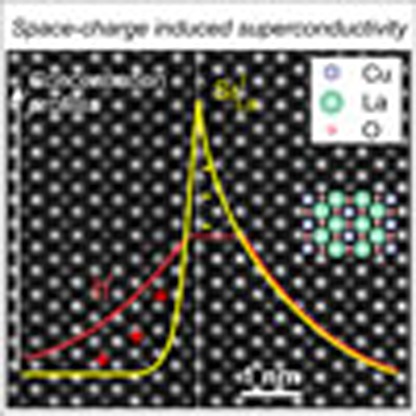

Interface effects in complex oxide heterostructures have received great attention because of the emergence of functional properties such as magnetism^1^,^2^,^3^,^4^, metallicity^5^,^6^,^7^, superconductivity^8^,^9^ and high-temperature superconductivity^10^ that are not shown by the phases individually. This rationale has been applied to ionic and mixed electronic/ionic conductors (‘heterogeneous doping')^11^,^12^,^13^, where this powerful technique, relying on the introduction of space-charge zones, has been proven to be on a par, if not superior, to the classic homogeneous (bulk) doping (see Supplementary Note 1). While superconducting electronic accumulation layers have been created at heterojunctions between two phases or at grain boundaries (for example refs 8, 9, 10, 14), the insertion of an atomic plane of defined interfacial charge (here substitutional insertion) yielding local high-temperature superconductivity has not been achieved so far. This is not only exciting in terms of generating space-dependent superconductivity without dopant disorder but also for purposeful functional structuring.

As well known, homogeneously doped La_2−*x*_Sr_*x*_CuO_4_, in which the resulting strontium (Sr) dopants (
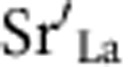
 in Kröger–Vink notation) give rise to a significant concentration of electron holes [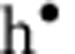], exhibits high-temperature superconductivity with critical temperatures (*T*_c_) up to ≈40 K (for the optimal doping *x=*0.16). For a higher doping level (‘overdoping'), *T*_c_ decreases and the structure eventually becomes metallic^15^,^16^. This is attributed to an interplay of electronic and structural reasons, since for a high Sr-content defect chemistry predicts a tendency to oxygen vacancy (
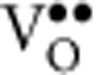
) compensation (because of double-positive charge), association between 
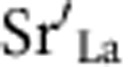
 and 
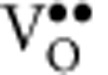
 and between 
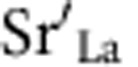
 and 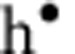 (refs 17, 18; see Supplementary Fig. 1 and Supplementary Note 2).

Unlike forming La_2−*x*_Sr_*x*_CuO_4_ solid solutions, we use here unconventional heterogeneous doping^11^,^12^,^13^ to enhance the hole concentration and thus trigger superconductivity. Inspired by the so-called delta-doping technique^19^,^20^,^21^, we replace single LaO planes with SrO ones, with the aim of creating two-dimensional (2D) and atomically confined charged layers within the undoped La_2_CuO_4_ matrix. So far, delta-doping in oxides referred to either the insertion of one (or more) complete unit cells of a doped material in a matrix of the same but undoped material^22^,^23^,^24^ or , in few cases dealing with the formation of 2D electronic gases or local modification of magnetic properties, the successful substitution of single atomic planes in perovskites (LaO for SrO planes^5^,^25^,^26^,^27^, SrO for GdO planes^28^). None of them used delta-doping for obtaining high-temperature superconductivity. Either comparatively thick layers were inserted^9^,^29^ or, in the few cases where 2D doping was employed^5^,^25^,^26^,^27^,^28^, superconductivity was not achieved.

Here the Sr-to-La substitution targets a single atomic layer of La_2_CuO_4_ and yields a layer of high effective negative charge density. Such an approach is expected to yield a local space-charge redistribution of 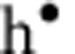 and mobile ionic charges^12^. While the possibility of cation redistribution deserves a closer inspection (see below), oxygen diffusion kinetics^18^ (Supplementary Fig. 2) certainly allow oxygen interstitials (
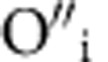
) and vacancies (
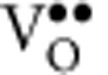
) to redistribute on the nm scale.

The rationale of this contribution is depicted in Fig. 1, which compares the two cases of homogeneously (zero-dimensional) doped with heterogeneously (2D) doped La_2_CuO_4_. While, in the first case (Fig. 1a,c), at equilibrium all positive (negative) defects are increased (depressed) on Sr-doping in correspondence to the randomly distributed Sr point defects, in the second case (Fig. 1b,d) the interfacial charge dictates the charge-wise similar but spatially very different behaviour in the space-charge zones. A SrO layer substituting one LaO layer in the La_2_CuO_4_ cell acts as a negatively charged interface, analogously to a charged grain-boundary case in a bicrystal^11^, yet with *a priori* known interfacial charge, resulting in an enhancement of the positively charged mobile defects (particularly holes) in the adjacent layers.

In the classical doping case, the probability of finding the excess hole in the local environment of a Sr-dopant is enhanced; yet, owing to the disorder, a high concentration of Cooper pairs in a given layer cannot build up. The transition to an ordered 2D situation corresponds to a transition from a Debye–Hückel to a Gouy–Chapman situation, in which the very high local interfacial charge, whose sign is known *a priori*, produces a sharp hole accumulation enabling measurability (see below), percolation efficiency and structurability.

Qualitatively, the effects of homogeneous and heterogeneous doping can be described by the following rules^30^:


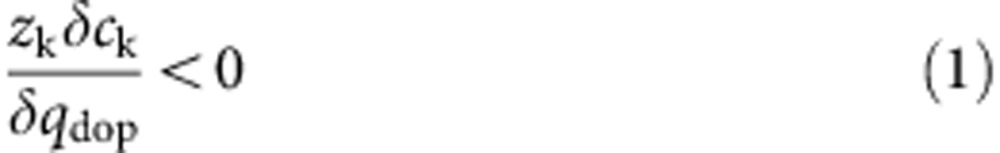


where *c*_k_ and *z*_k_ are the concentration of the charge carrier *k* and its charge number, respectively, and *q*_dop_ the charge of the dopant. This expression indicates that on homogeneous addition of acceptors into a host material (for strontium substituting lanthanum *q*_dop_<0)) the concentrations of all positively (negatively) charged carriers *c*_k_ are increased (decreased), as long as they are mobile and can attain distribution equilibrium. This follows from charge conservation and the electroneutrality of the disorder reactions. In the case of heterogeneous doping :


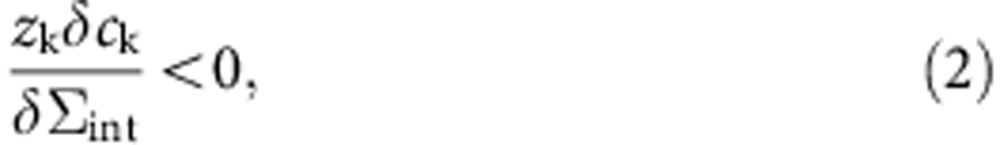


where ∑_int_ represents the effective charge density of the 2D dopant. Equation (2) follows from the fact that all charge carriers perceive the same electrical potential^30^.

By employing atomic-layer-by-layer oxide molecular beam epitaxy technique (ALL-oxide MBE), which allows for a definition of the nominal composition of each atomic layer^31^,^32^, we synthesized more than 100 epitaxial superlattices, in which single LaO layers in the La_2_CuO_4_ structure were substituted by SrO layers with the desired periodicity (see Supplementary Methods and Supplementary Figs 3–5 for preparation and structural characterization). Ignoring oxygen defects, the resulting composition of the heterostructures is described as (Fig. 2a):


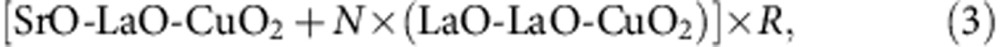


with *R* representing the number of superlattice unit repetitions and *N* the spacing between the SrO layers (expressed in number of constituting La_2_CuO_4_ units, which corresponds to half a unit cell).

The two sides of each SrO plane (the downward side facing the substrate and the upward side oriented towards the free surface) exhibit remarkably different properties. At the downward side, a clear decoupling between hole and Sr concentration, whose profile is here abrupt, is detected. This finding proves space-charge effect as the key mechanism yielding superconductivity. At the upward side instead, where Sr redistribution occurs, superconductivity arises because of conventional homogeneous doping.

## Results

### Resistivity measurements reveal confined superconductivity

The in-plane resistivity data collected from a set of samples having comparable total thicknesses (≈400 Å) and different spacing *N* between subsequent SrO layers is shown in Fig. 2b. Notably, the samples exhibit high-*T*_c_ superconductivity, with *T*_c_ (Fig. 2c) reaching a maximum value of ∼35 K for 5≤*N*≤9 (see Supplementary Methods as well as Supplementary Figs 6 and 7 for the definition and analysis of *T*_c_). For higher spacing *N*, *T*_c_ saturates at only ≈25 K and the in-plane resistivity drastically increases. This lowering of the *T*_c_ for large *N* is connected with a lower orthorhombicity^32^, a trend that is observed also in the case of conventional doping. These results suggest that conducting layers are formed in the proximity of each SrO layer. For short distances between two subsequent SrO layers (namely for *N*<5), *T*_c_ and resistivity decrease, suggesting ‘overdoping' or structural distortions. For *N*≤1, a non-superconducting phase is formed.

### Abrupt Sr profile at the downward side of the interface

To elucidate the local microstructure and composition, high-resolution transmission electron microscopy, together with analytical characterizations, was systematically performed employing aberration-corrected scanning transmission electron microscopy (TEM, see also Supplementary Methods). The high-angle annular dark-field (HAADF) micrograph in Fig. 3a reveals that the superlattice exhibits perfect epitaxy, illustrating the absence of any extended structural defects such as misfit dislocations or antiphase boundaries. A high-resolution micrograph is presented in Fig. 3e, showing the atomic arrangement in greater detail. In the intensity profile obtained from the HAADF image (averaged perpendicularly to the growth direction), the intensity drop is connected with the Sr-containing layers (compare Fig. 3a,b). It is noteworthy that, such a change of intensity involves more than a single atomic plane indicating a certain Sr redistribution into the La_2_CuO_4_ matrix. Highly spatial-resolved spectroscopic analyses (Fig. 3c,d) provide more precise information revealing a pronouncedly asymmetric character of the Sr profile: virtually abrupt at the side facing the substrate (downward side) with an extent of 0.9±0.2 nm and smeared over 2.3±0.4 nm at the upward side, a feature that will be explained below. Results from energy-dispersive X-ray spectroscopy (EDXS) allows for a quantitative estimation of the La^3+^/Sr^2+^ concentration ratio (Fig. 3c and Supplementary Fig. 8). The maximum estimated ratio, obtained by averaging the maximum intensities of the different peaks, is 0.21±0.02. Electron energy-loss spectroscopy (EELS; Fig. 3d) provides further robust evidence of the asymmetric Sr distribution, ensuring single atomic layer resolution (step size≈2 Å)^33^,^34^.

By averaging the different EELS Sr-L_2,3_ intensity profiles from several Sr-containing atomic slabs, and applying an appropriate scaling factor to satisfy the nominal overall composition of the compound, one can accurately define the Sr level (*x*) that can be assigned to each ‘constituting block' (namely a single CuO_2_ plane and the two surrounding (La,Sr)O layers) in proximity of the layer where Sr was initially inserted (see Fig. 3f and also Supplementary Methods, Supplementary Figs 9 and 10). Obviously, we have realized a rather abrupt profile, but only at the downward side, while at the other side (upward) there is a pronounced redistribution of the aliovalent cation. According to our rationale, we expect the space-charge mode to be active at the downward side and a usual homogeneous mode at the upward side (*cf.* Supplementary Note 3 and Supplementary Fig. 11). Taking both these mechanisms into account, the expected hole profile is sketched in Fig. 4.

### Zn tomography specifies layer-dependent superconductivity

Further complementary tests relying on the Zn-doping tomography technique^21^ were carried out on structures containing one single SrO layer (Fig. 5a). The method consists of substituting ∼3% of Cu with Zn and thus suppressing *T*_c_ of specific CuO_2_ planes (Supplementary Fig. 12 and Supplementary Methods). In this way, spatial information is available, which allows us to identify which particular CuO_2_ planes are responsible for high-*T*_c_ superconductivity. To determine the contribution to superconductivity of each single CuO_2_ plane at the downward side of the interface, we proceeded as follows: all the CuO_2_ planes at the upward side were Zn-doped but on the downward side selected single CuO_2_ planes were Zn-doped and the electrical transport properties measured. Analogously, the same procedure was applied to determine the contribution of each single CuO_2_ layer of the upward side, after that the *P=−*2 CuO_2_ plane of the downward side had been Zn-doped.

**Figure 1:**
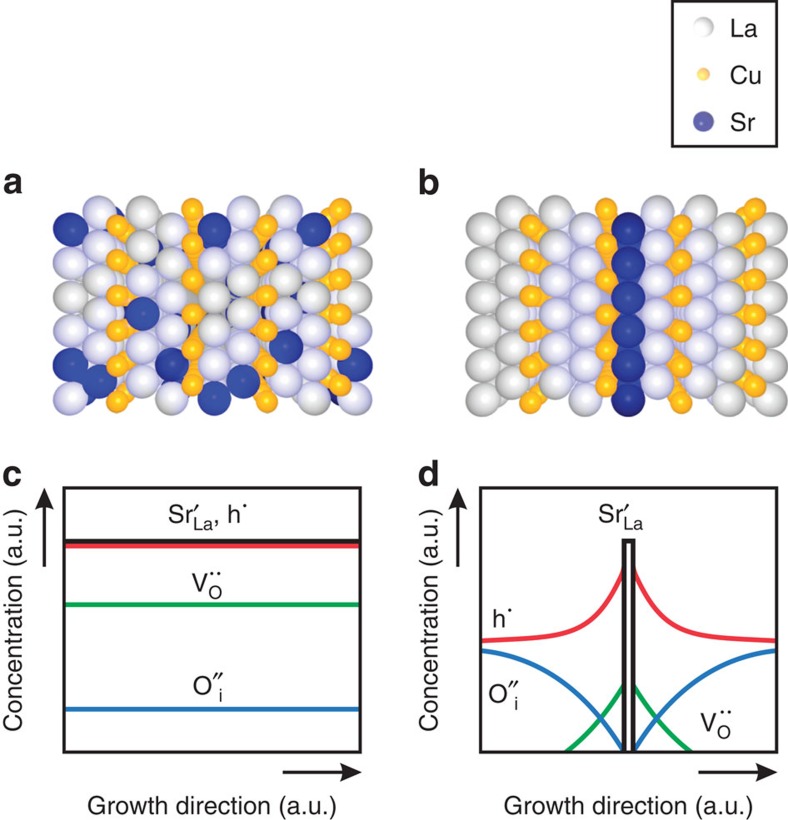
Homogeneous versus heterogeneous doping for lanthanum cuprate. The crystal structures for two cases are schematically represented in **a**,**b**, respectively (the oxygen ions are omitted for clarity). In **a**, 
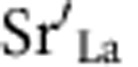
 are randomly distributed as point defects, whereas in **b** they are ordered into a crystallographic plane forming a negatively charged layer. In both cases, in proximity of the 
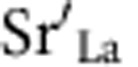
 defects, structural as well as compositional (that is, formation of compensating charge defects) modifications are expected. The resulting defect concentration profiles are depicted in **c**,**d** for the zero-dimensional and the two-dimensional doping case, respectively. The disordered spatial distribution of 
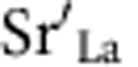
 defects leads, in the first, to a homogeneous increase in hole (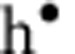) and oxygen vacancy 
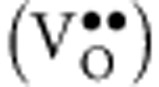
 concentration, and to a depressed negatively charged oxygen interstitial 
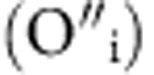
 concentration with respect to the undoped material. In **d**, as a consequence of two-dimensional doping, the formation of a sharp hole accumulation layer with the space-charge layer is predicted.

**Figure 2:**
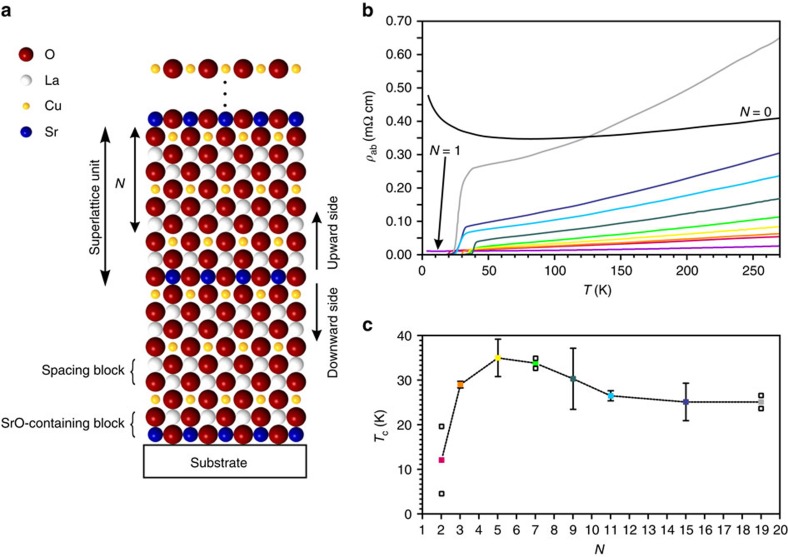
Electrical properties of two-dimensionally doped La_2_CuO_4_ superlattices. In **a**, a sketch of the structure is represented. Starting from the La_2_CuO_4_ structure, superlattices are formed by regularly replacing single layers of LaO with SrO planes. The general formula can be written as: [SrO-LaO-CuO_2_+*N* × (LaO-LaO-CuO_2_)] × *R.* The periodicity *N* is defined as the number of spacing blocks between two SrO-containing blocks, whereas *R* indicates the number of superlattice units. (**b**) Representative in-plane resistivity measurements for superlattices with different spacing *N*, epitaxially grown along the *c*-direction on LaSrAlO_4_ (001) substrates. Each spacing is marked with a different colour as defined in **c**, in which the solid square symbols represent the value of the critical temperature *T*_c_ plotted as a function of *N*. Here the error bars show the s.d. of the *T*_c_ values obtained for a set of samples having the same composition. For those compositions (value of *N*), in which a set of two samples was considered for the determination of the average critical temperature, both *T*_c_ data points are shown in the diagram (open square symbols). The uncertainty in the definition of the *T*_c_ value can be ascribed to normal sample-to-sample variations due to, for example, light stoichiometry offset, substrate roughness and strain.

**Figure 3:**
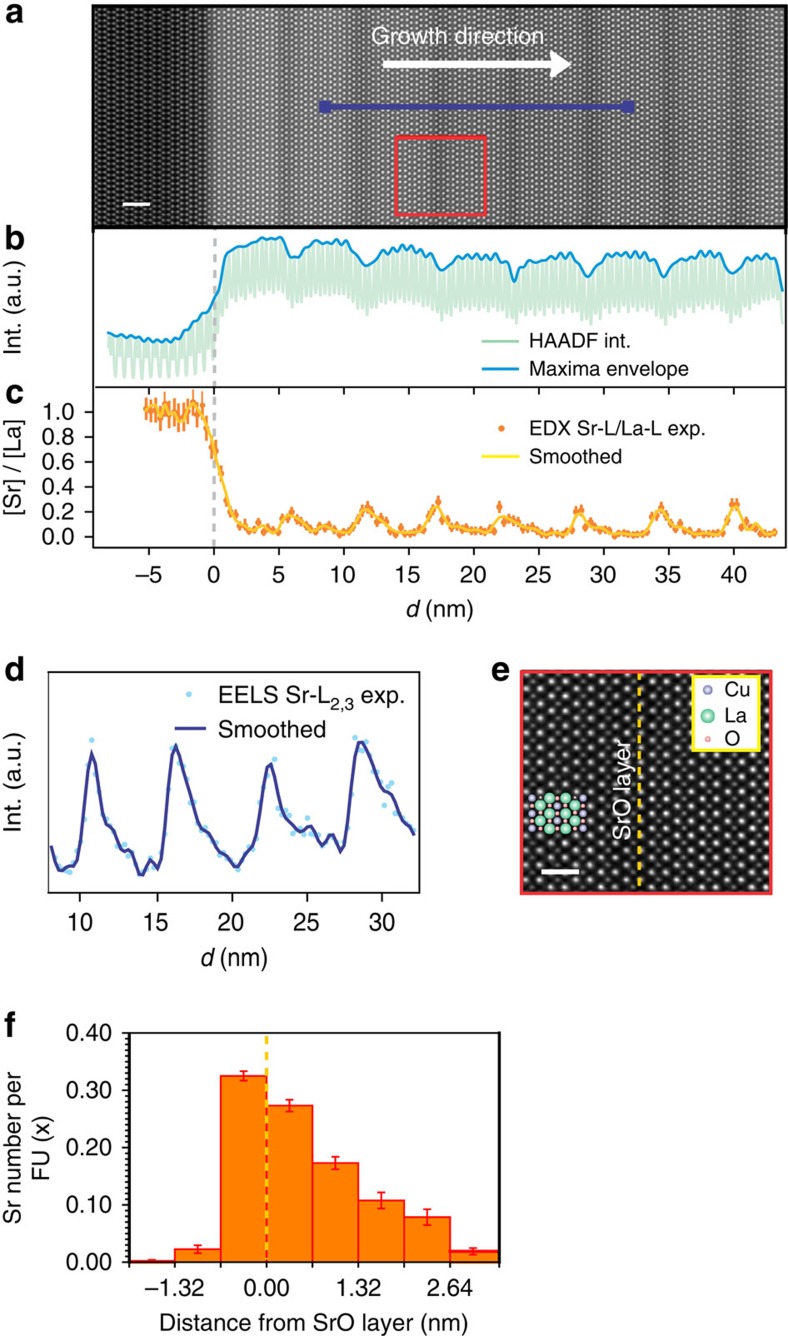
High-resolution transmission electron microscopy (HRTEM) analysis of cationic distribution in two-dimensional doped La_2_CuO_4_. (**a**) HAADF–STEM image of two-dimensionally doped La_2_CuO_4_ showing the microstructure of a superlattice (*R=*8, *N=*7) grown on the LaSrAlO_4_ (001) substrate. The alternation of brighter and darker areas reflects the superlattice structure, in which Sr-doped regions (dark) are separated by undoped La_2_CuO_4_ (bright). This is clearly shown by the maxima envelope of the image-intensity profile, integrated perpendicular to the growth direction (**b**, dark blue line). Scale bar, 2 nm. In **b**, the intensity oscillations of the intensity profile due to the different contrast of each atomic layer (green line) can be seen. A magnified image of the region highlighted in red in **a** is shown in **e**, in which the dotted yellow line corresponds to the layer having maximum Sr content. Scale bar, 1 nm. (**c**) [Sr]/[La] ratio, extracted from an EDXS line scan across the region shown in **a**. Asymmetric Sr distribution, smeared in the growth direction, is detectable. Sr-L and La-L lines were used for quantifying the Sr concentration, and the integrated signals of Sr and La were calibrated using the substrate region where [La]/[Sr] is equal to unity (see Supplementary Fig. 8 for the net counts of Sr-L and La-L EDXS lines). Here error bars are the square root of the intensity. A similar Sr asymmetric profile results from the integration of the Sr-L_2,3_ EELS line profiles, as shown in **d**, which has been acquired across four Sr-containing layers (blue line in **a**). Here the error bars (square root of the intensity) are smaller than the symbols. From the EELS analysis, the average Sr number per formula unit FU (*x* in La_2−*x*_Sr_*x*_CuO_4_), for each (La,Sr)O-CuO_2_-(La,Sr)O ‘constituting block' in proximity of the Sr-containing layers, as depicted in **f**, was obtained (the s.d. is represented by the error bars).

The results are summarized in Fig. 5b, in which *T*_c_ obtained after having placed Zn in the different CuO_2_ planes is shown (see also Supplementary Fig. 13). The two sides exhibit similar *T*_c_ (orange and green areas in Fig. 5b for downward and upward sides, respectively), in contrast to what one would expect according to a simple homogeneous doping picture (see section above). Moreover, while no remarkable effect coming from Zn substitution in single CuO_2_ planes could be observed for the upward side (consistent with a homogeneous doping situation predicting several CuO_2_ planes to be superconducting, *cf.* Fig. 3f), for the downward side, we found *T*_c_ to stay almost unaffected when Zn was put in each plane *P* (−4≤*P*≤0), except for *P=*−2. In this case, the insertion of Zn leads to a drastic *T*_c_ reduction (*T*_c_<4 K), allowing us to identify this CuO_2_ plane (*P=*−2) as the main source of high-*T*_c_ superconductivity of the downward side. Owing to the very low local Sr content (*x* ≈0.02), this undoubtedly demonstrates that superconductivity at the downward side does not arise from homogeneous doping. Rather, one has to refer to the heterogeneous case as sketched in Fig. 1d, which results in a hole accumulation, up to the optimal for superconductivity, for *P=*−2. Please note again that, in all the samples, which were prepared to investigate the role of the downward side of the interface by Zn tomography, all the *P≥*1 planes were doped by Zn with the purpose of suppressing any contribution to *T*_c_ stemming from the upward side.

### Electrical properties of the La_2_CuO_4_/SrO bilayer

To confirm the minor role of the La^3+^/Sr^2+^ intermixing in the definition of the electrical properties at the downward side, we also considered a structure consisting of a bilayer of La_2_CuO_4_ and a 10-unit-cell-thick SrO film (Fig. 6a). Here the growth dynamics for the downward side follows a similar sequence compared with the structure depicted in Fig. 5a, with the only difference that several SrO layers are deposited in succession. If cation intermixing was responsible for the observed superconductivity at the backward side of the SrO planes, then we would expect to detect superconductivity also in this bilayer as long as we can neglect serious accommodation problems of the replaced La. Nevertheless, no sign of metallicity or superconductivity could be found (Fig. 6b, Supplementary Note 4 and Supplementary Fig. 14), confirming the relevance space-charge effects induced by the heterogeneous doping mechanism.

### STEM–EELS confirms the predicted hole profile

**Figure 4:**
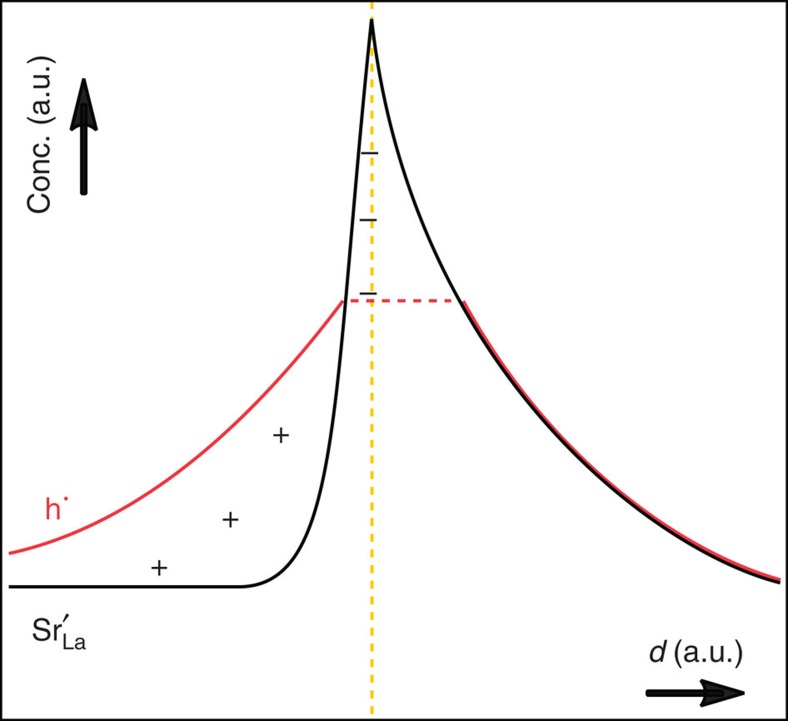
Expected hole profile in the Sr-doped region. As a consequence of Sr distribution, the expected hole profile is sketched (see also Supplementary Fig. 11). Hole and Sr spatial distributions are depicted in red and black, respectively. While on the right-hand (upward) side the hole concentration follows the Sr profile (cf. Fig. 1c), on the left-hand (downward) side a space-charge situation occurs, resulting in an accumulation layer profile (cf. Fig. 1d). The dotted yellow line represents the nominal position of the SrO layer.

**Figure 5:**
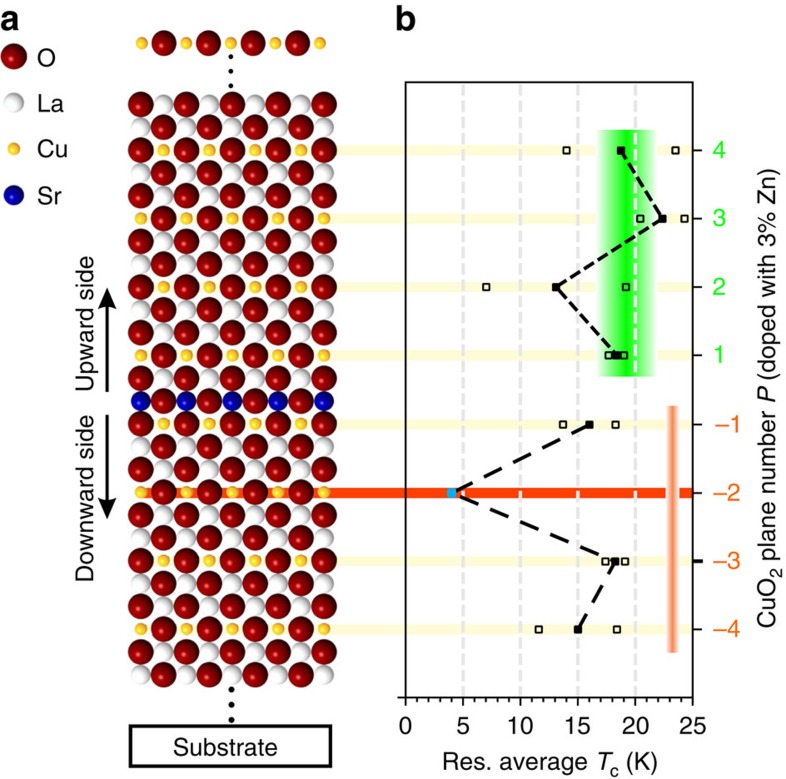
Zn tomography on two-dimensionally doped La_2_CuO_4_. (**a**) Sketch of the symmetric structure investigated by Zn tomography (the SrO atoms are depicted in blue). By means of Zn-doping, one can suppress the superconducting critical temperature of selected CuO_2_ planes in proximity of the SrO layer, thus mapping *T*_c_ with atomic layer precision. The results are shown in **b**, where the average residual *T*_c_ (closed squares) is plotted as a function of the Zn-doped CuO_2_ plane *P*. One can see that doping by Zn the *P=−*2 plane (marked in red) leads to a substantial reduction of *T*_c_, indicating that this plane is mainly responsible for superconductivity at the downward side. The indicated *T*_c_ value (closed light blue square) has to be considered as an upper limit, since the critical temperature for the samples was below the low limit for our measurements (4 K). As for the upward side, doping by Zn single CuO_2_ planes does not have a pronounced effect, indicating that several planes are responsible for *T*_c_. Reference *T*_c_ for the upward and downward sides are indicated in green and orange, respectively. Open squares indicate the single data points.

**Figure 6:**
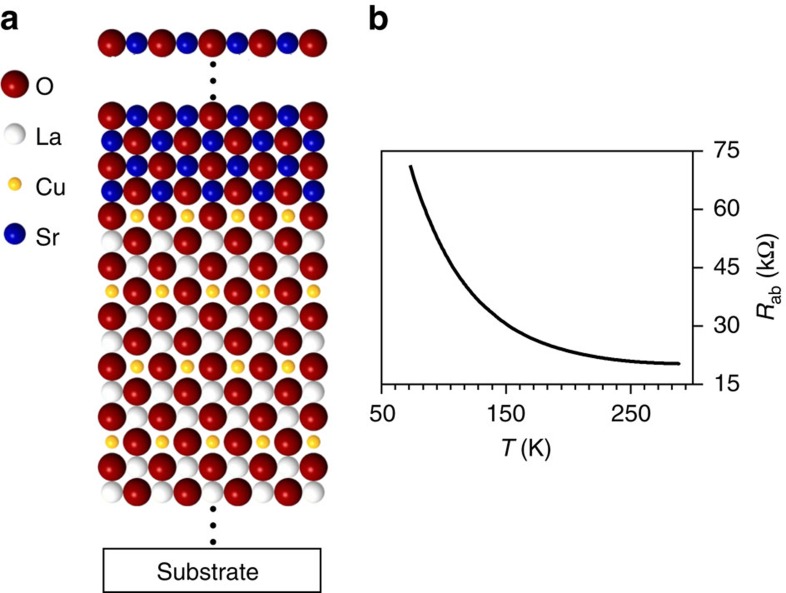
Electrical behaviour of the La_2_CuO_4_/SrO bilayer. (**a**) Sketch of the La_2_CuO_4_/SrO bilayer structure. Unlike the structure represented in Fig. 5a, where the SrO is an effective atomic layer in the La_2_CuO_4_ structure, thus being negatively charged with respect to the replaced LaO layer, here it forms a neutral second phase (a thick SrO layer). The electrical properties of such a structure (15 u.c. (unit cell) La_2_CuO_4_/10 u.c. SrO), exhibiting semiconducting behaviour, are illustrated in **b**.

**Figure 7:**
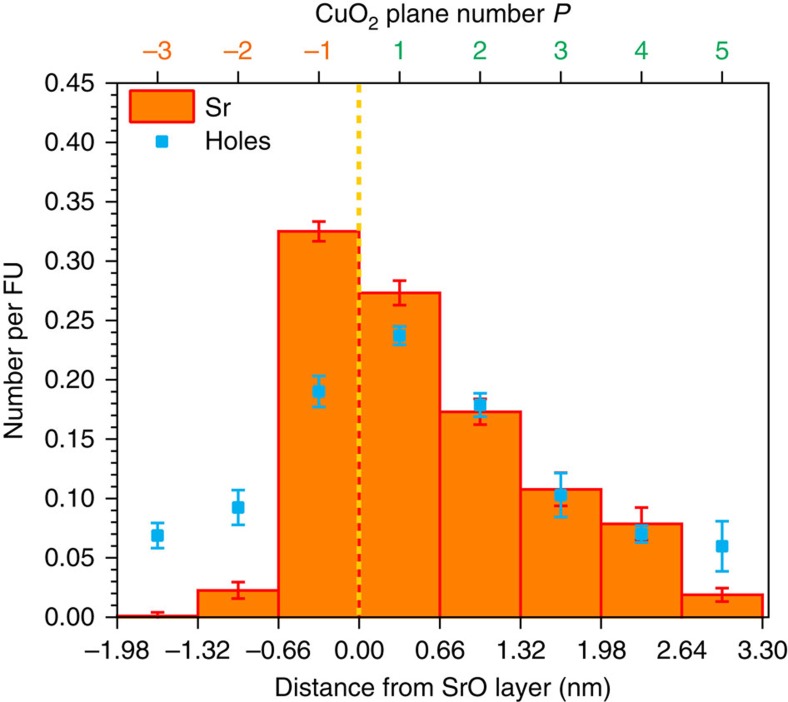
Comparison of Sr and hole concentration profiles as obtained by STEM–EELS. The number of holes per formula unit assigned to each constituting block (La,Sr)O-CuO_2_-(La,Sr)O for a structure having spacing *N=*7, as obtained by the O–K-edge EELS analysis (see Supplementary Fig. 10 and Supplementary Methods—s.d. indicated by error bars) is reported here in comparison with the Sr profile as depicted in Fig. 3f. The CuO_2_ plane numbering is made in agreement with Fig. 5. Note the decoupling between the two profiles at the downward side interface, and the far-reaching agreement with Fig. 4.

A further nice corroboration of the findings described above stems from the intensity profile of the oxygen-K-edge pre-peak^35^, which is attributed to electronic transitions from the O_1s_-core level to hole states with *p* symmetry in the valence band (*cf.* Supplementary Fig. 15). As these data are averaged over each (La,Sr)O-CuO_2_-(La,Sr)O-constituting block, we compare it with the averaged Sr profile obtained from EELS analysis in Fig. 3f. The scaling was performed to satisfy global electroneutrality (ignoring oxygen point defects—see Supplementary Methods). The results are shown in Fig. 7. One clearly recognizes local electroneutrality on the right-hand side (upward side) and space-charge accumulation on the left-hand side (downward side). The qualitative agreement with the prediction given in Fig. 4 is remarkable. In addition, the absolute value of the hole concentration [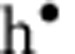] is reasonable: to plane *P=*−2 corresponds a hole concentration ≈0.1 per CuO_2_ plane, which for single phase thin films on LaSrAlO_4_ (001) substrates has *T*_c_≈25 K (refs 15, 21), as indeed estimated by the Zn-doping experiment (*cf*. Fig. 5b). This agreement in absolute numbers may be partially coincidental because of possible structure distortions^36^, and to the fact that the high-*T*_c_ behaviour in space-charge zones may differ from the bulk situation.

## Discussion

The most fascinating of the above described results regards the realization of a space-dependent superconductivity induced, as a consequence of a space-charge effect, by the Sr abrupt profile at the downward side of the SrO plane. While EDXS and EELS Sr-L_2,3_ intensity profiles reveal the Sr concentration profile to abruptly decrease even in close proximity of the SrO plane, the analysis of the pre-peak of the oxygen-K-edge indicates a substantial accumulation of 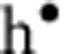 within the same region. Electrical transport measurements carried out according to the Zn tomography technique corroborate these findings by demonstrating that the CuO_2_ plane-labelled *P=*−2 is the main source of high-*T*_c_ superconductivity at the downward side. Such hole accumulation (decoupled from Sr), due to the excess negative charge of the SrO layer, leads to a highly space-dependent *T*_c_. The decay length of the hole profile is consistent with the bulk defect chemistry of pure La_2_CuO_4_ governed by 2
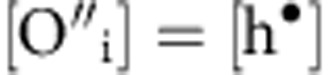
17.

It is noteworthy that, while in the case of homogeneous doping a rather large volume is necessary for inducing percolating superconductivity^22^,^37^, a space-charge-induced two-dimensional layer serves for highest volume concentration in terms of Cooper pairs. (As to the influence of dimensionality on superconductivity *cf*. ref. 38). Moreover, heterogeneous doping offers the advantage of experimentally probing the space-charge-induced superconductivity by means of different and complementary techniques, including STEM–EELS (scanning transmission electron microscopy–EELS) and Zn-doping tomography. In the literature, there is only one report on a possible decoupling between 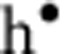 and 
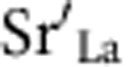
 at the interface between metallic La_2−*x*_Sr_*x*_CuO_4_ and insulating La_2_CuO_4_ (ref. 10), yet also at the upward side (in growth direction) of the interface where we rather observe Sr redistribution.

At this side, the situation is dominated by non-negligible dopant redistribution leading to 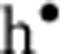 locally neutralizing diffused 
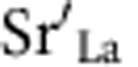
 point defects. Such mixing effects have been seen in MBE-grown δ-doped semiconductors^19^,^39^ and, in some cases, in PLD-grown complex oxide systems^40^,^41^. Its occurrence is due to the specific kinetics of the atomic-layer-by-layer deposition process (Supplementary Methods), assuming intermixing to occur only between the last atomic layers on the film surface and the incoming species of high-kinetic energy. This may also be connected with the observed roughness of the film (Supplementary Fig. 16) and the high surface atomic mobility during growth. In such a nonequilibrium situation, cation exchange occurs in the growth direction only^42^, whereas it is limited in the downward direction because of sluggish diffusion. Finally, dopant segregation to the surface of the heterostructure^43^, which has been theoretically predicted for La_2_CuO_4_ (ref. 44), provides an additional, mainly energetic, driving force for the formation of such a Sr profile.

In summary, this study demonstrates the significance of heterogeneous doping in the field of superconductivity. It represents not simply an alternative to the homogenous doping approach but rather it offers a series of unique features. First, in the case of 2D doping, one can tune the local charge distribution (and thus local superconductivity) without affecting the chemical composition of the host material and without introducing local crystallographic disorder resulting from the dopant solid solution. This allows for a clear space-charge effect and for structuring the matrix material at wish, that is, we are able to insert active layers in the matrix and control their number, position and relative distance. From an operational point of view, it is certainly desired and will be attempted in the future to arrive at a two-sided abruptness of such profiles rather than an asymmetric situation.

Second, one can induce layer-dependent high-temperature superconductivity, while conventional doping leads to a homogeneous situation. Third, such superconductivity induced by a 2D charge distribution yields the formation of a layer of high Cooper pair concentration unlike the case of induction by homogeneously distributed dopant atoms. Fourth, the technique may be applied to situations where the solubility of the dopant is limited (and conventional doping is not possible) and/or a large volume is required for a sufficient percolation.

Last, our findings can be used to shed light on the influence of dimensionality on superconductivity^38^ but also on the importance of typically neglected effects of defect chemistry and ionic mobility in the field of high-temperature superconductivity.

## Methods

### ALL-oxide MBE synthesis

Thin films of 2D Sr-doped lanthanum cuprate were grown on LaSrAlO_4_ (001) substrates (Crystec GmbH), using ozone-assisted MBE^31^,^32^. The deposition conditions used for synthesizing the samples are temperature *T*≈620 °C and pressure *p*≈2.5 × 10^−5 ^ Torr (of mixed ozone, molecular and atomic oxygen). It is worth noting that, after the growth, all the samples were cooled under vacuum starting from a temperature *T=*200°C, which ensures that no contribution to electrical conductivity of La_2_CuO_4_ can be ascribed to extrinsic doping by interstitial oxygen (*cf.* ref. 32). A more detailed description of the growth method and sample structural characterization can be found in the Supplementary Methods.

### Conductivity measurements

Electrical properties of the films were probed using direct current (DC)-electrical conductivity measurements as a function of temperature, using a Keithley 2,000 multimeter and a Keithley 2,400 sourcemeter implemented in a home-built system. The measurements were performed by warming up the sample from ≈4 K to room temperature, with a rate<0.1 K s^−1^, applied by a motorized dipstick whose motion was controlled via the customized LABVIEW software. Pt contacts were deposited on sample corners by sputtering, and measurements were carried out in a four-point (Van Der Pauw) configuration. To minimize any contribution due to possible sample inhomogeneities, several measurements were carried out on the same sample on rotation.

### Electron microscopy and spectroscopy

The cross-section specimens for STEM experiments were thinned to electron transparency by tripod polishing down to ≈10 μm and argon ion beam milling in a stage cooled with liquid nitrogen. The electron microscopy and spectroscopy measurement were performed on a JEOL ARM 200CF microscope equipped with a cold field-emission electron source, a probe C_s_ corrector, a large solid-angle SDD-type JEOL Centurio EDX detector and a Gatan GIF Quantum ERS spectrometer. The microscope was operated at 200 kV, a semiconvergence angle (*α*) of 21 mrad, giving rise to a probe size of 0.8 Å (1 Å for the analytical analysis). Collection angle (109–270 mrad) was used to obtain the HAADF images. A collection semi-angle (*β*) of 68.5 mrad was used for EELS measurements. The STEM analysis was carried out at the specimen region where thickness is below 30 nm (*t/λ*<0.4 measured by low loss EELS using a log-ratio method). A detailed analysis about EELS data evaluation can be found in the Supplementary Methods and Supplementary Figs 9 and 10.

## Additional information

**How to cite this article:** Baiutti, F. *et al*. High-temperature superconductivity in space-charge regions of lanthanum cuprate induced by two-dimensional doping. *Nat. Commun.* 6:8586 doi: 10.1038/ncomms9586 (2015).

## Supplementary Material

Supplementary Information Supplementary Figures 1-16, Supplementary Notes 1-4, Supplementary Methods and Supplementary References
