# Supplementary material for: High-temperature superconductivity in space-charge regions of lanthanum cuprate induced by two-dimensional doping
Source: Nat Commun. 2015 Oct 20;6:8586. doi: 10.1038/ncomms9586 (PMC4634214; doi:10.1038/ncomms9586)
Supplement: Supplementary Information — Supplementary Figures 1-16, Supplementary Notes 1-4, Supplementary Methods and Supplementary References [file ncomms9586-s1.pdf]

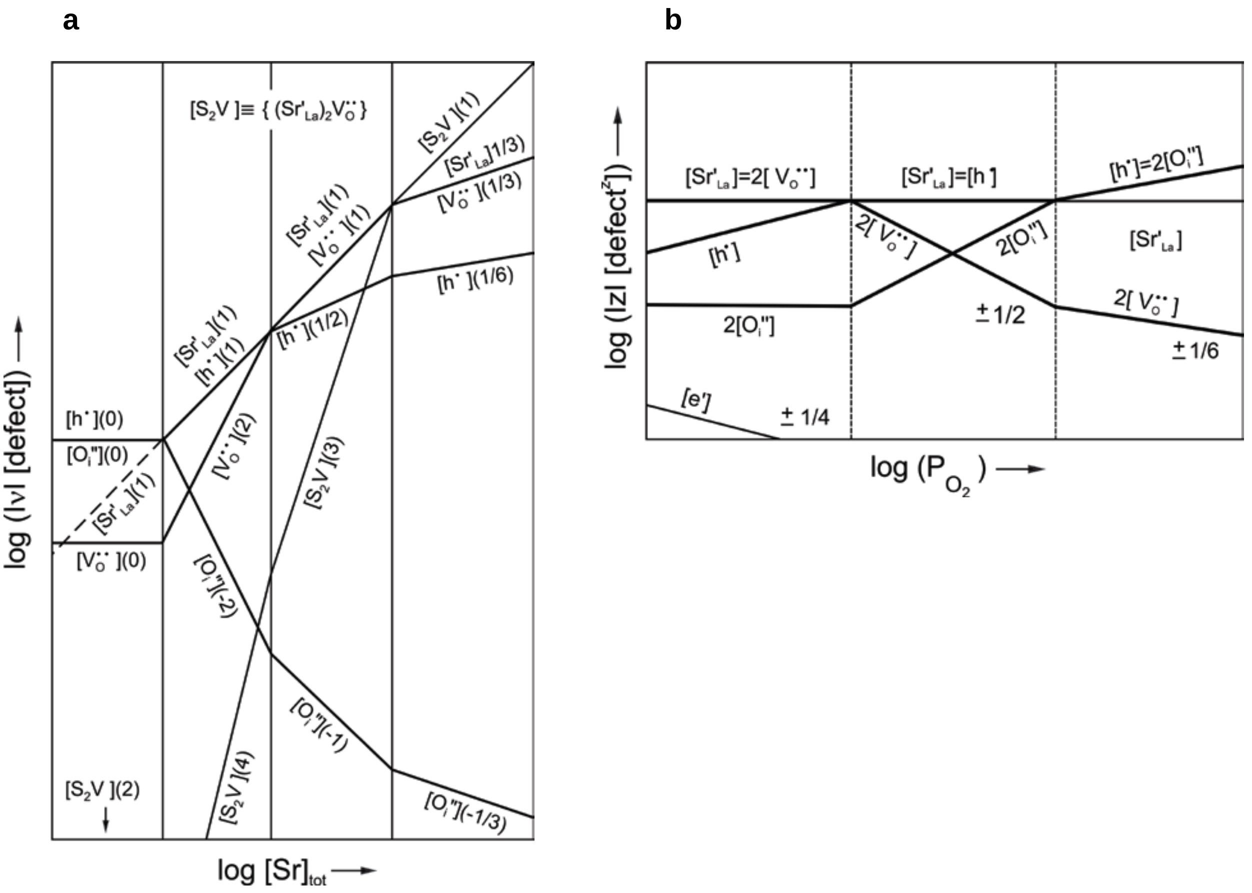

Supplementary Figure 1. Dependence of charge carrier concentrations on Sr-doping (a) and oxygen partial pressure (b) under homogeneous equilibrium conditions. They are taken from Refs. 1,2 and are reproduced with permission. As |z| in panel (b), the parameter |ν| in the ordinate labeling of panel (a) gives the absolute value of the charge in the case of ionized defects; in case of S_2V, however, it gives the Sr content per formula unit.

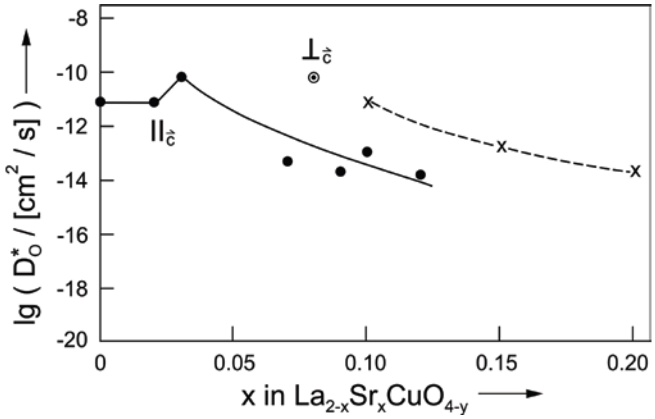

Supplementary Figure 2. Oxygen tracer diffusion coefficients at T = 873 K. The figure is taken from Ref. 2 and reproduced with permission. In Ref. 3 also the T-dependence is discussed.

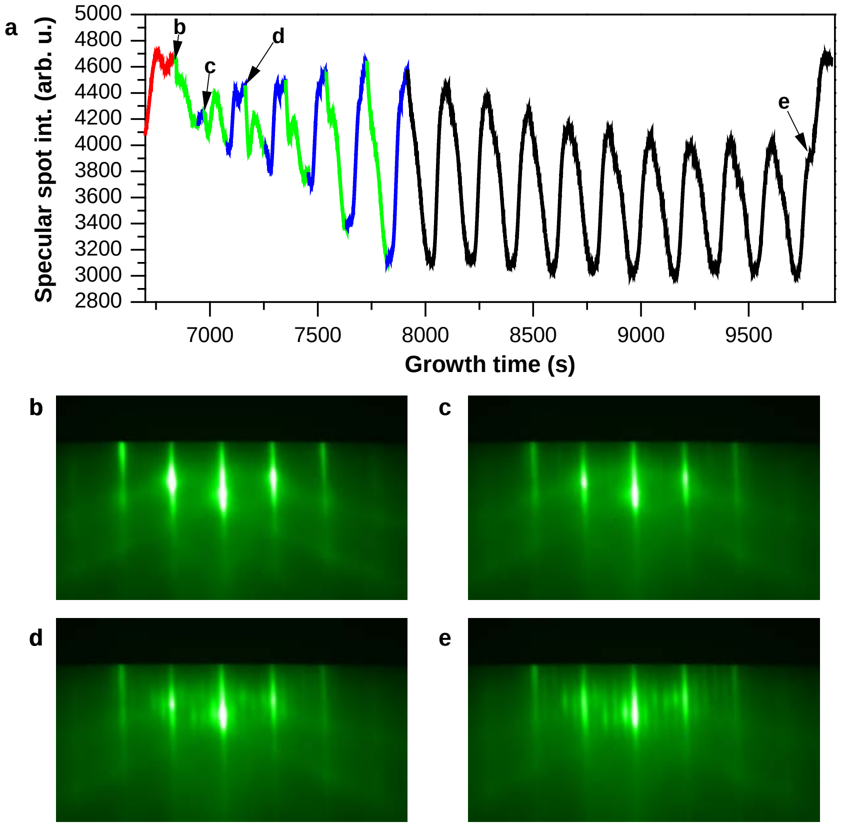

Supplementary Figure 3. RHEED in-situ analysis during the growth of a two-dimensionally doped superlattice period [(SrO-LaO-CuO_2 + 15 x (LaO-LaO-CuO_2)]. (a) Specular spot intensity evolution. The different stages of atomic layer deposition are marked with different colours for the first layers (SrO = red, LaO = green, CuO_2 = blue). Diffraction patterns along the (100) direction, recorded at the end of the deposition of the SrO layer (b), and of different CuO_2 layers ((c) to (e)) are also reported.

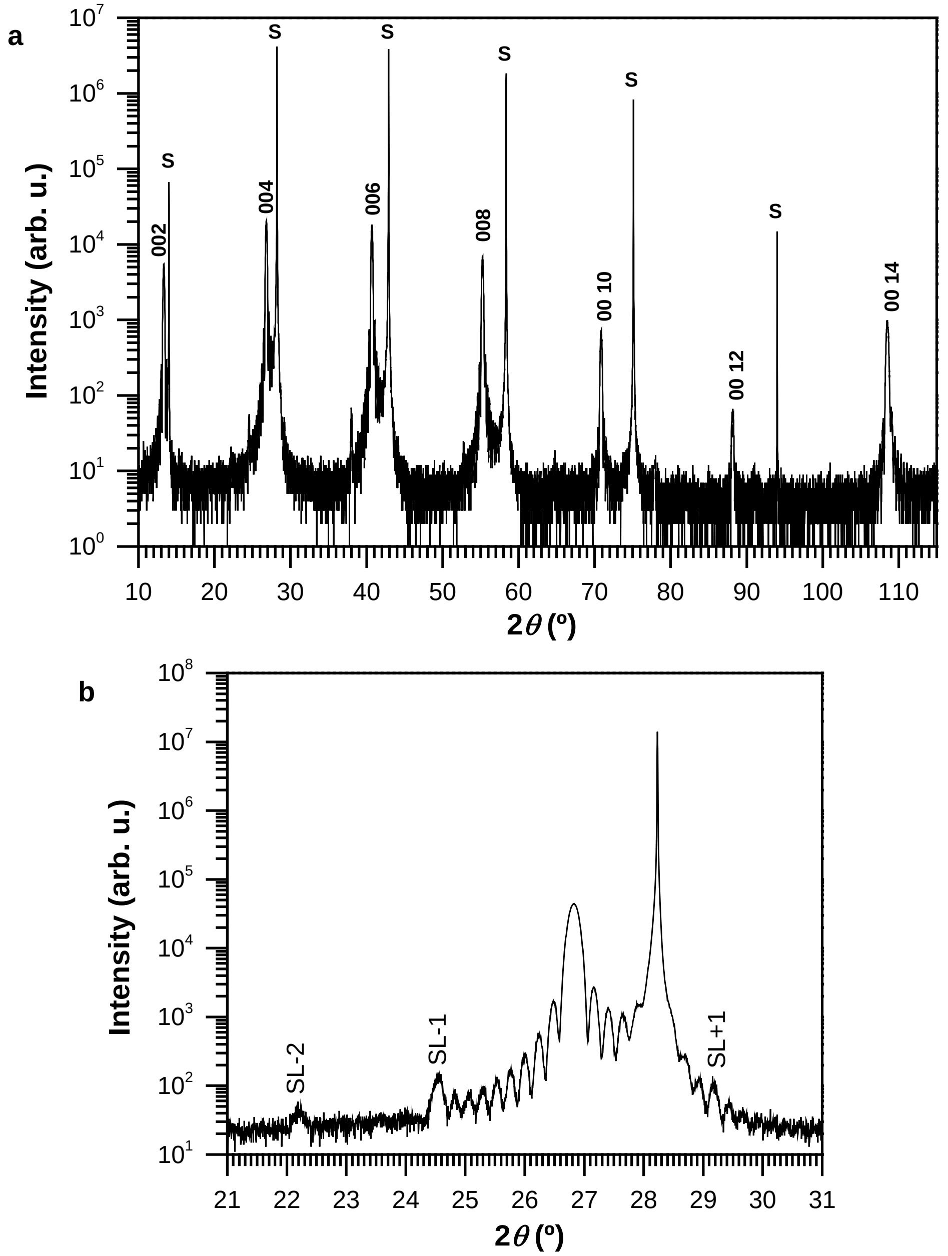

Supplementary Figure 4. High-resolution XRD patterns of a two-dimensionally doped La_2CuO_4 superlattice. The structure is [(SrO-LaO-CuO_2) + 5 x (LaO-LaO-CuO_2)] x 10 on $LaSrAlO_4$ (001) substrate. (a) 10º $\leq 2\theta \leq$ 115º full scan (substrate diffraction peaks are indicated as S). (b) Magnification around the (004) diffraction peak, showing finite thickness fringes and superlattice n^{th}-order satellite peaks (SL-n).

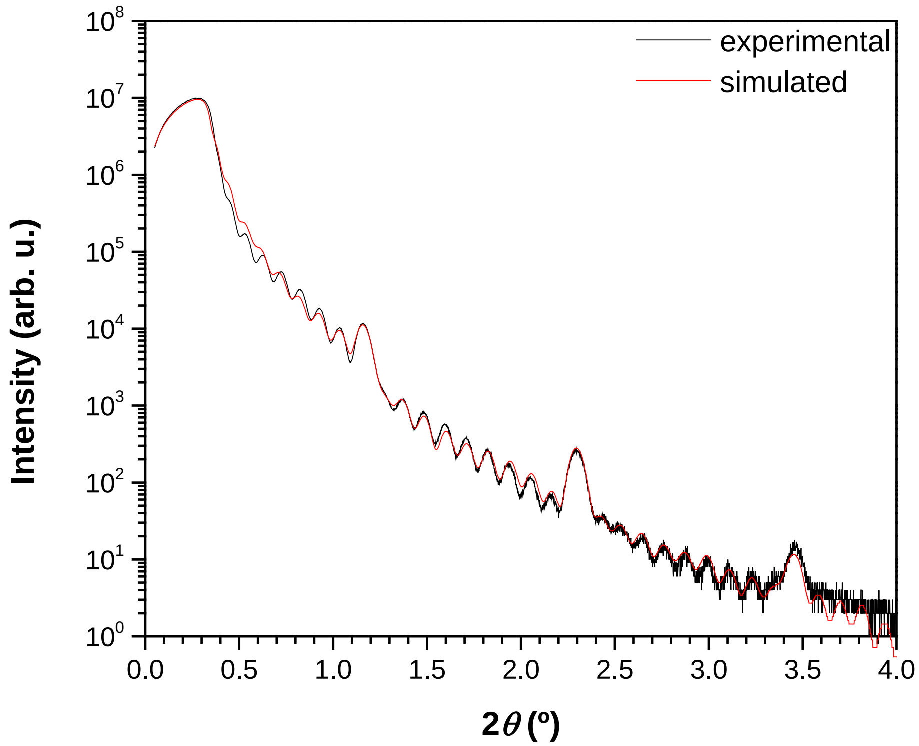

Supplementary Figure 5. Low-angle XRR scan of a thin film for a two-dimensionally doped La_2CuO_4 superlattice. The superlattice composition is [(SrO-LaO-CuO_2) + 5 x (LaO-LaO-CuO_2)] x 10 on $LaSrAlO_4$ (001) substrate. The data simulation for the reported sample results in an average period thickness of 38.56 Å, to be compared with an expected thickness of 39.84 Å.

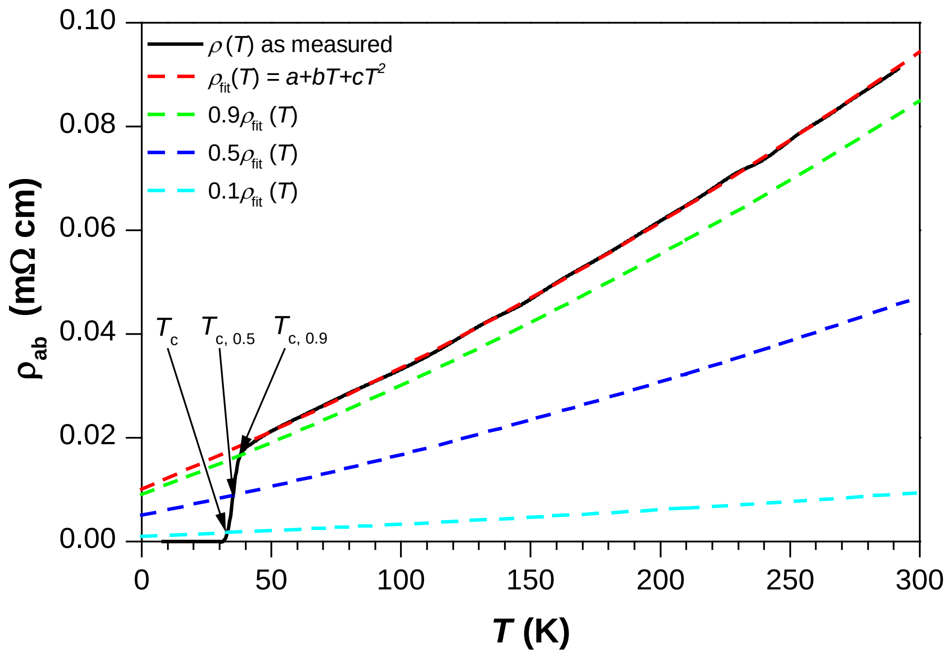

Supplementary Figure 6. Definition of the superconducting transition temperature values. T_c, $T_{c,0.5}$ and $T_{c,0.5}$ are defined at the intercept between the experimental resistivity curve $\rho(T)$ and the fitting function ρ_{fit} (T), appropriately scaled.

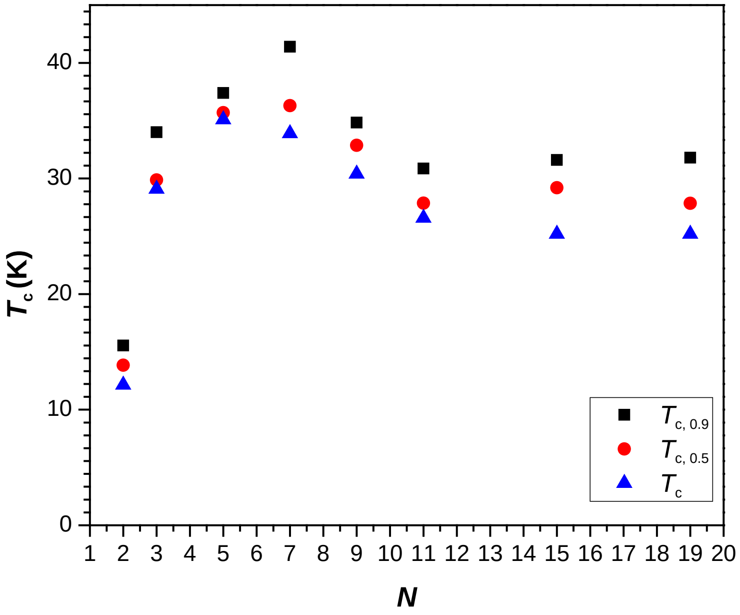

Supplementary Figure 7. Average superconducting transition temperature $T_{c,0.9}$, $T_{c,0.5}$ and T_c as a function of the spacing N, for two-dimensionally doped La_2CuO_4 having general formula [(SrO-LaO-CuO_2) + N x (LaO-LaO-CuO_2)] x R.

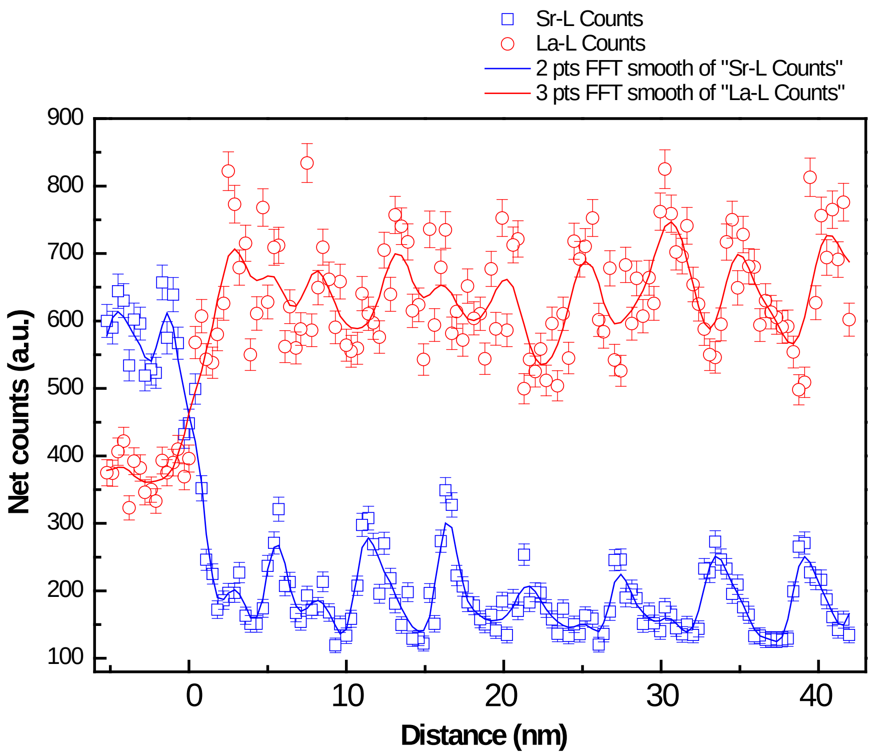

Supplementary Figure 8. Net counts of Sr-L and La-L EDX lines. The error bars are calculated as square root of the intensity.

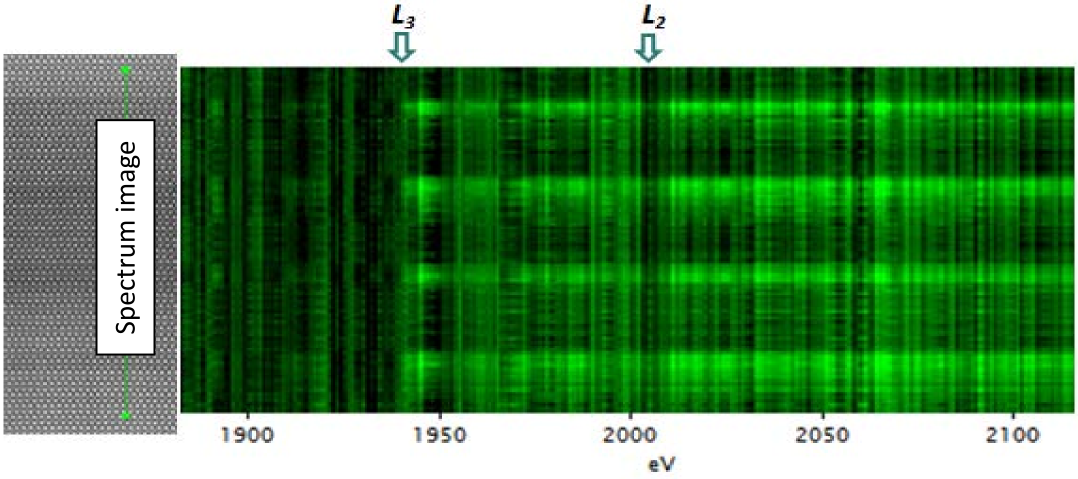

Supplementary Figure 9. EELS spectrum image (line-scan) of the Sr-$L_{2,3}$ edge after background subtraction. Left: survey image showing the line along which the EELS line scan was acquired. Right: EELS spectrum image. The asymmetric Sr profile is readily visible in the raw data.

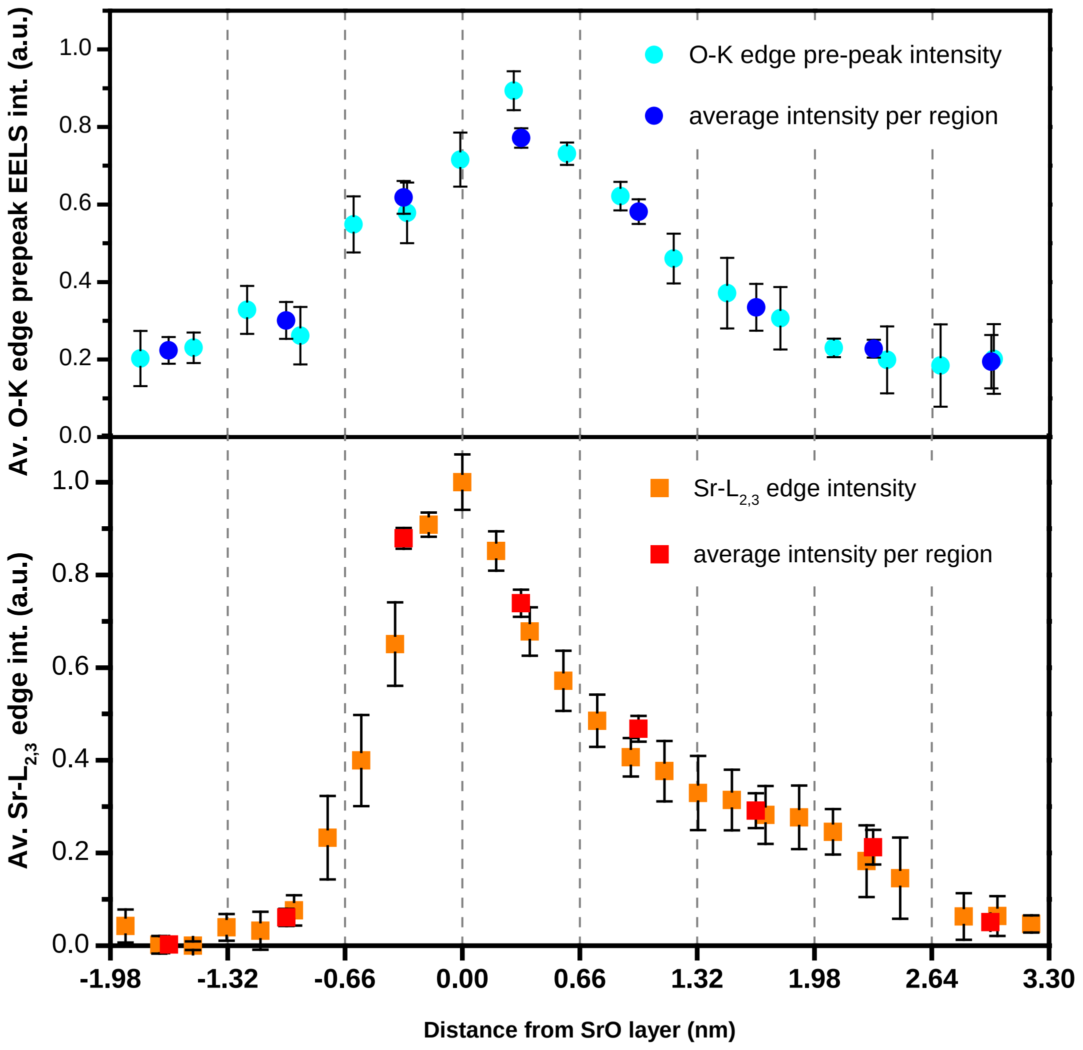

Supplementary Figure 10. EELS data analysis of Sr and hole concentrations. The experimental EELS data for holes and Sr, obtained by averaging the intensity profiles of different line scans, are indicated in light blue and orange, respectively (standard error is indicated by error bars). The dashed lines divide the regions corresponding to each (La,Sr)O-CuO_2-(La,Sr)O constituting block. The data obtained as weighted mean for each region are shown in dark blue (for holes) and red (for Sr). For holes, the intensity is expressed as the result of the subtraction of the reference spectrum (referring to undoped La_2CuO_4, see Supplementary Figure 15).

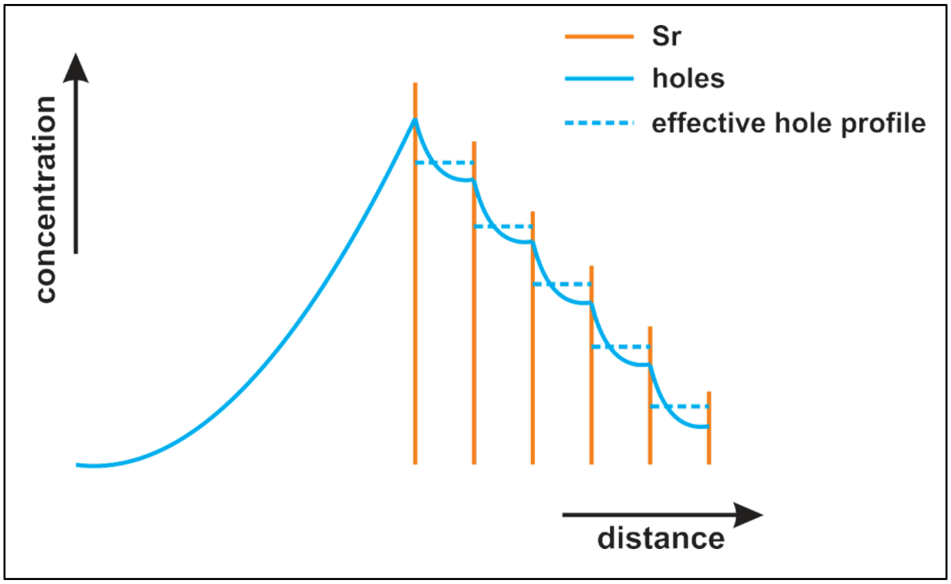

Supplementary Figure 11. Hole concentration profile expected in a continuum approximation, as a consequence of a Sr distribution as in two-dimensionally doped La_2CuO_4.

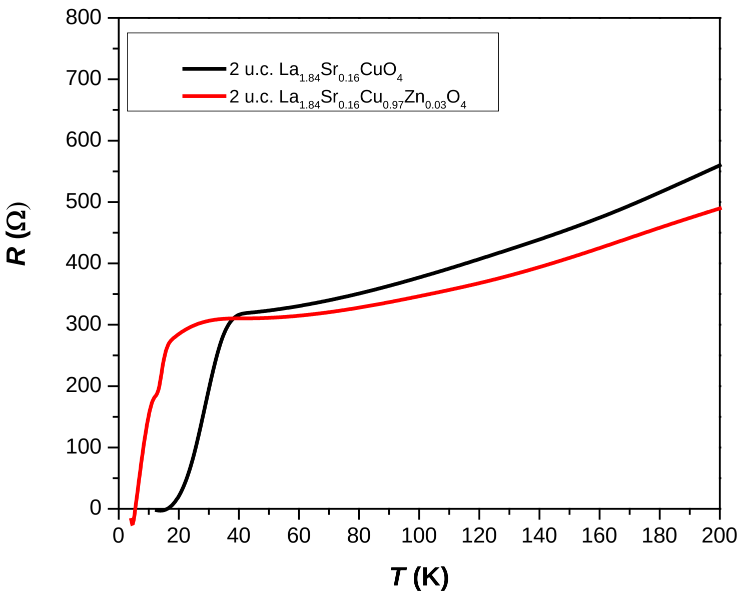

Supplementary Figure 12. *R* vs *T* for ultrathin films of single phase $La_{1.84}Sr_{0.16}CuO_4$ (black curve) and $La_{1.84}Sr_{0.16}Cu_{0.97}Zn_{0.03}O_4$ (red curve).

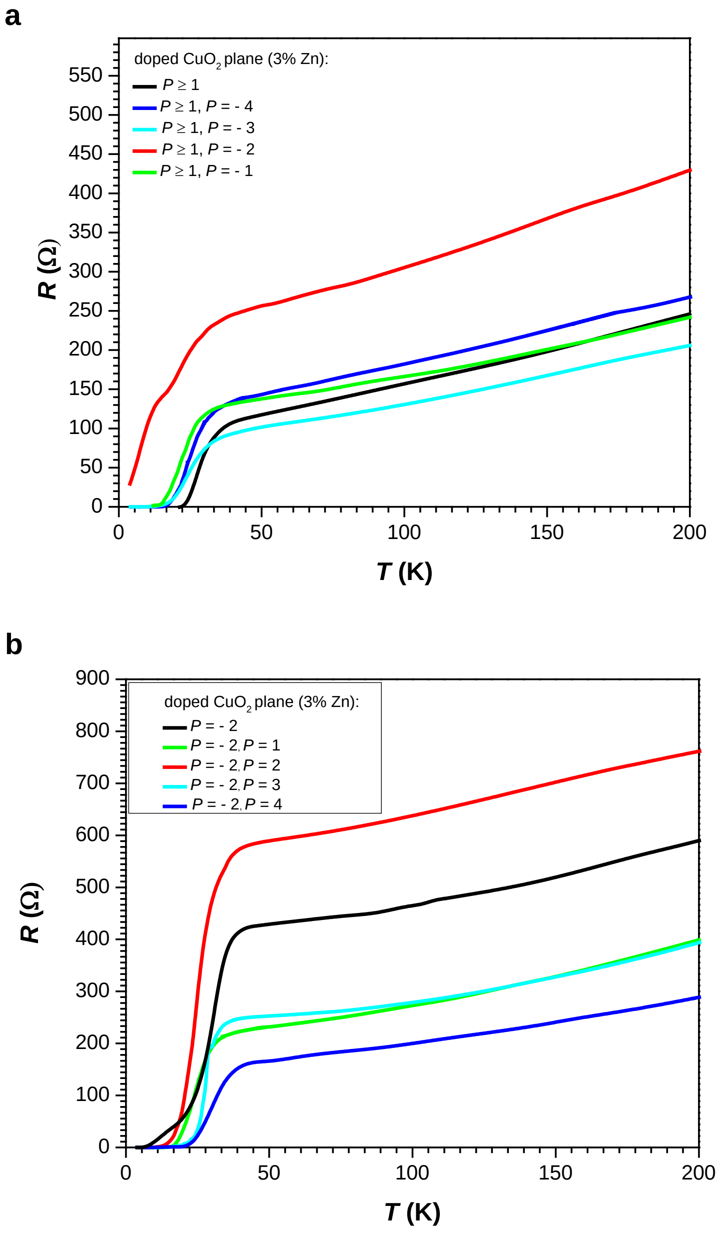

Supplementary Figure 13. *R* vs *T* for two-dimensionally doped La_2CuO_4 upon Zn-doping. In panel (a), results from the study of the downward side (b) results from the upward side analysis.

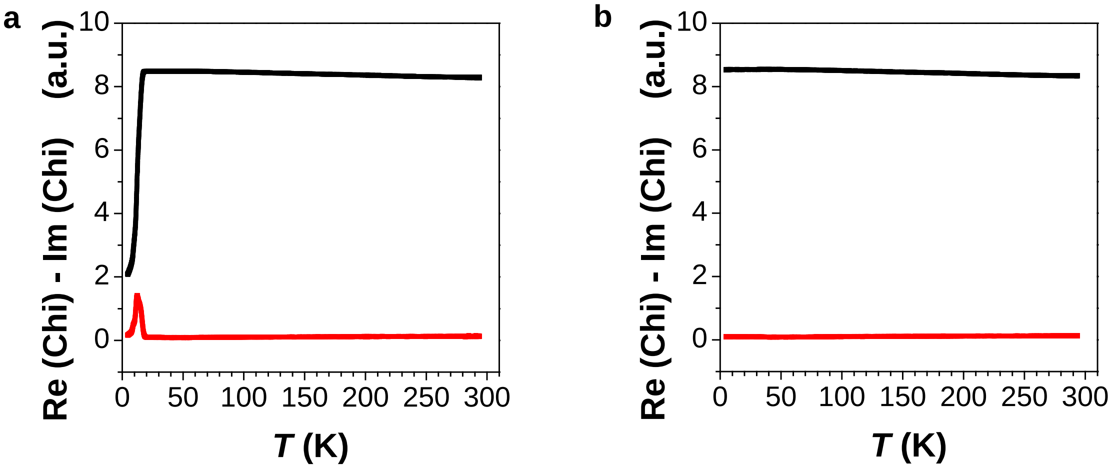

Supplementary Figure 14. Magnetic susceptibility measurements obtained from a two-dimensionally doped La_2CuO_4 structure, in which only a single SrO plane is present (a), compared with the results stemming from the measurement of a La_2CuO_4 / SrO bilayer (b)**.**

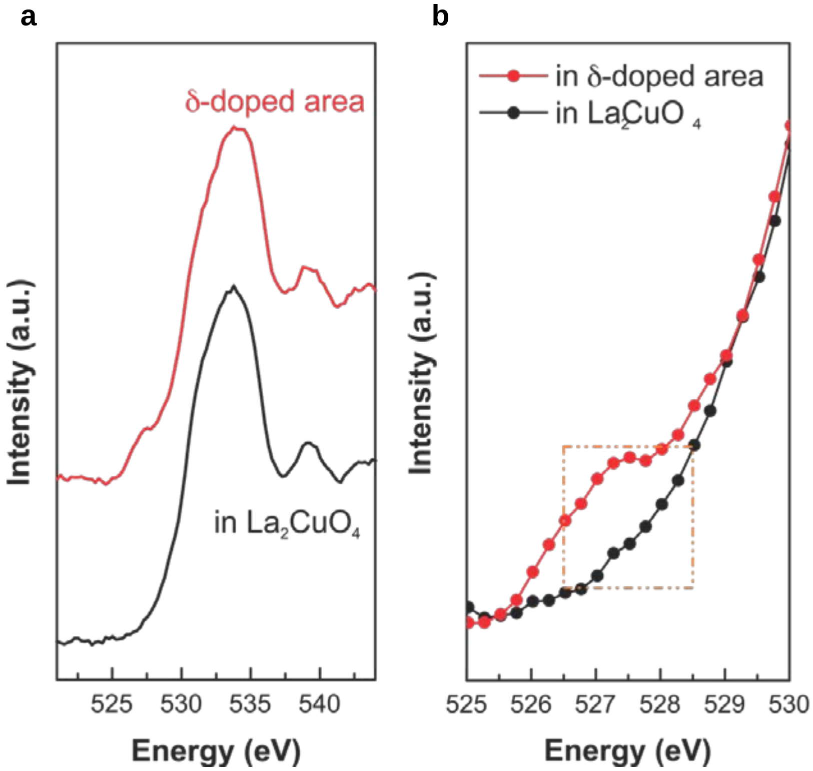

Supplementary Figure 15. (a) Oxygen-K edge from two-dimensionally doped La_2CuO_4 (red), and from La_2CuO_4 (black), respectively, showing clear changes in the fine structure. In the hole-enriched region, a pre-peak can be seen. (b) Details for counting the net O pre-edge counts, by comparing the spectrum with pre-edge peak with a reference spectrum in La_2CuO_4 without pre-edge peak.

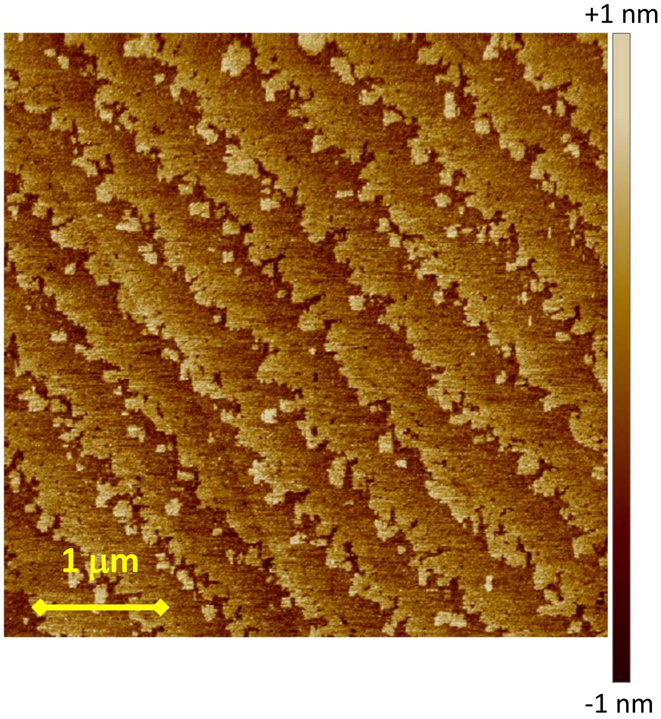

Supplementary Figure 16. Atomic force microscopy of a film surface (area = 25 μm^2). R_{ms} roughness is as small as 0.3 nm.

Supplementary Note 1
Homogeneous vs. heterogeneous doping

It is worth noting that in the context of nanoionics the terms homogeneous and heterogeneous doping are well defined, viz. in the way the terms are used in this text. In the semiconductor literature heterogeneous doping is sometimes used unprecisely for the classical doping mode in the case of inhomogeneous dopant distribution. Note that the term “heterogeneous” refers to spatially different sites of different local free energy (e.g. different phases). In the case of heterogeneous doping, one can distinguish between one- (e.g. insertion of dislocations), two- (e.g. introduction of layers as here) and three-dimensional doping (e.g. introductions of particles). The random introduction of zero-dimensional structural elements (point defects) is equivalent to (classical) homogeneous doping.

Supplementary Note 2
La_2CuO_4 and $La_{2-x}Sr_xCuO_4$ defect chemistry[1,2,3]

High-temperature superconductivity in lanthanum cuprate occurs upon hole doping $h^\bullet$. In case of pure La_2CuO_4, this can be achieved by accommodation of interstitial oxygen O_i'' occupying an empty interstitial site V_i^x:

$$\frac{1}{2}O_2 + V_i^x \leftrightarrow O_i'' + 2h^{\bullet}$$

The electroneutrality condition reads:

$$2[O_i''] = [h^{\bullet}]$$

Another way of increasing the hole concentration is via acceptor doping (typically Sr substituting La Sr_{La}'). In this case, the acceptor dopant incorporation can be written as:

$$2SrO + 2La_{La}^x + O_O^x \rightarrow La_2O_3 + 2Sr_{La}' + V_O^{\bullet\bullet}$$

The electroneutrality equation reads:

$$[Sr_{La}'] = [h^{\bullet}] + 2\,[V_O^{\bullet\bullet}]$$

In case of high doping level, a tendency to vacancy compensation is predicted, as well as the formation of defect associates (e.g. $\{(Sr_{La}')_2V_O^{\bullet\bullet}\}$).

Supplementary Figure 1a displays the variation of defect concentration for $La_{2-x}Sr_xCuO_4$ on Sr-doping at fixed oxygen partial pressure. In Supplementary Figure 1b, the expected defect concentrations are given as a function of oxygen partial pressure at fixed Sr-content. Note that the middle P_{O_2} region refers to pure hole-compensation. Both defect diagrams refer to conditions where the interaction with oxygen is in equilibrium.

Supplementary Figure 2 describes the oxygen tracer diffusion coefficient D_O^* for $La_{2-x}Sr_xCuO_4$ as a function of Sr concentration x. The initial increase of D_O^* upon Sr-doping, followed by a drastic decrease in oxygen diffusivity, is consistent with a diffusion model via oxygen vacancy mechanism[20], taking also into account ordering interactions. Note also the substantial anisotropy of the tracer diffusion coefficient.

Supplementary Note 3
Expected hole distribution in a continuum approximation

The different mechanisms of hole distribution at the downward and upward sides of the interface (two-dimensional and zero-dimensional doping, respectively), are presented in a continuum approximation (i.e. ignoring hole localization on CuO_2 planes) in Supplementary Figure 11. While on the left-hand side (downward) we face a semi-infinite space charge zone, on the right-hand side (upward) we have a sequence of finite, almost flat, contributions. On this basis, Figure 4 is constructed.

Supplementary Note 4
Superconductivity in La_2CuO_4/SrO bilayer

In order to further investigate the superconducting properties of the La_2CuO_4 / SrO bilayer, additional measurements of magnetic susceptibility[4] in a parallel geometry were carried out using a home-built two-coil Mutual Inductance setup which includes a Stanford Research System SR850 DSP lock-in amplifier and Agilent 34401A multimeter. One could think that the insulating electrical behavior of the bilayer as shown in Figure 6b, measured applying electrical contacts on the surface of SrO, could in principle be

ascribed to the top layer (SrO) acting as an insulator towards any metallic phase underneath. Magnetic susceptibility measurements based on the Meissner effect are able to detect superconducting transitions irrespective of this. In Supplementary Figure 14, we compare the results of such measurements in case of two-dimensionally doped La_2CuO_4 as depicted in Figure 5a (Supplementary Figure 14a), compared with the La_2CuO_4 / SrO bilayer as depicted in Figure 6a (Supplementary Figure 14b): in the first, the superconducting transition is clearly visible, whereas no signal variation comes from the bilayer. This provides an additional robust proof of the different properties of the two structures.

Supplementary Methods

Sample growth

High-quality epitaxial thin film films of Sr two-dimensionally doped La_2CuO_4, in form of superlattices, have been grown on $LaSrAlO_4$ (001) substrates (Crystec GmbH) using a custom designed Oxide Molecular Beam Epitaxy (Oxide-MBE) built by DCA Instruments. The system is suitable for the atomic-layer-by-layer growth method, based on the deposition of a single atomic layer at a time. Sample growth is based on thermal evaporation of atomic species from metal sources and subsequent oxidation and phase formation on the substrate. Each source flux is carefully controlled by electro-pneumatic shutters, whose timing is first defined by rate calibration via a quartz crystal monitoring system (QCM) and is then optimized during the process. The main in-situ monitoring tool is provided by Reflection High Energy Electron Diffraction (RHEED). A more detailed description of the system can be found in Ref. 32.

In Supplementary Figure 3, the typical (100) direction RHEED diffraction analysis, recorded during the growth of a single superlattice period Sr two-dimensionally doped La_2CuO_4 sample, is shown. In Supplementary Figure 3a, specular spot intensity is reported as a function of growth time, while RHEED patterns during different stages of the deposition are shown in Supplementary Figures 3b to 3e. One can see that, after the deposition of the SrO atomic layer (the corresponding specular spot time evolution is marked in red in Supplementary Figure 3a), one does not observe the presence of any diffraction signal except the one that is attributed to the perovskite-type phase typical of La_2CuO_4 (Supplementary Figure 3b). This suggests that no secondary phases such as rock-salt SrO islands are formed during growth (cf. Reference 5). Rather, SrO is deposited as atomic layer in the La_2CuO_4 structure. The oscillation amplitude obtained during the deposition of the subsequent LaO (green line in Supplementary Figure 3a) and CuO_2 (blue line) atomic layers, initially reduced, is restored at the distance of about 4 constituting blocks, i.e. 2 unit cells, from the SrO layer. Notably, such a distance corresponds to the Sr / La intermixing width in the growth direction as detected by HR-TEM analysis, further confirming that this process mainly occurs during the growth rather than once the structure is formed. The diffraction pattern observed at the end of the deposition of the CuO_2 layer shows the typical reconstruction feature of La_2CuO_4, i.e. 4 extra-bands between the main diffraction streaks, suggesting the presence of a surface superstructure (cf. supplementary information of Reference 6) (Supplementary Figures 3d, 3e). This feature is less pronounced in Supplementary Figure 3c, which represents the diffraction pattern observed at the end of the deposition of the two-dimensionally doped constituting block, suggesting an increased crystallographic disorder.

Structural characterization

Ex-situ structural characterization was performed including high-resolution X-ray diffraction (HR-XRD) using a Bruker D8 Diffractometer (Cu-$K_{\alpha 1}$ = 1.5406 Å), X-ray reflectivity (XRR), obtained using a

Panalytical Empyrean Diffractometer as well as Atomic Force Microscopy (AFM) using Digital Instruments with nanoscope III controller. 2θ–ω HR-XRD scan is shown in Supplementary Figure 4. $10^{\circ} \leq 2\theta \leq 115^{\circ}$ full scan (Supplementary Figure 4a) demonstrates that films are oriented along the *c*-direction, as shown by the diffraction main peaks; high crystallinity is indicated by the presence of XRD peaks of the film up to the (00 14) diffraction order. Supplementary Figure 4b shows the magnified (004) diffraction peak, which exhibits finite thickness fringes and superlattice satellite peaks. In Supplementary Figure 5**,** typical low-angle XRR for one of the synthesized superlattices is reported. Thickness and superlattice-peak fringes are clearly observed. Simulations have been performed using the software by Panalytical (based on the Parratt formalism for reflectivity, see Ref. 7) in order to fit the experimental data and verify period thicknesses. The results show that the thickness deriving from the best fitting curve ("simulated") is in excellent agreement with the nominal superlattice structure. In Supplementary Figure 16, a typical AFM micrograph is shown. Terraces, deriving from the substrate miscut angle ($\approx 0.1^{\circ}$), are clearly visible and indicate the 2D layer-by-layer growth of the film. Moreover, no precipitates of secondary phases can be observed. Root mean square roughness (R_{ms}) is comparable with the roughness of the substrate ($\approx$ 0.3 nm).

Electrical properties

Analysis of the resistivity data has been performed in agreement with Ref. 6, supplementary information. Resistivity curves $\rho(T)$ have been fitted using a quadratic polynomial function $\rho_{fit}(T) = a+bT+cT^2$ in the temperature interval 50 K < T < 270 K. After plotting $0.9\rho_{fit}(T)$, $0.5\rho_{fit}(T)$, $0.1\rho_{fit}(T)$, three different superconducting transition temperature values ($T_{c,0.9}$, $T_{c,0.5}$ and T_c) have been defined at the intercept between $\rho(T)$ and the three curves (Supplementary Figure 6). In Supplementary Figure 7, all $T_{c,\,0.9}$, $T_{c,\,0.5}$ and T_c are plotted as a function of superlattice spacing N.

Electron microscopy and spectroscopy

In Supplementary Figure 8, the net counts intensity for the Sr-L and the La-L EDX lines is reported. After calibration, obtained using the substrate region, the [Sr] / [La] ratio for the structure was obtained (see main text, Figure 3c). EELS linescan of the Sr-$L_{2,3}$ edge after background subtraction is shown in Supplementary Figure 9.

The O-K near-edge fine structure (Supplementary Figure 15) as well as the Sr distribution have been investigated by EELS. Supplementary Figure 10a presents the O-K edge as recorded in two-dimensionally doped La_2CuO_4 (red) (N = 7) and in undoped La_2CuO_4 (black) (taken as a reference). A pre-edge feature at around 528 eV is clearly seen in the spectrum acquired in the two-dimensionally doped La_2CuO_4 in the hole-enriched region, which is attributed to electronic transitions from O1s-core-level to hole states with *p* symmetry in the valence band[8,9]. To quantify the net intensity of this pre-edge peak, we subtracted the reference spectrum from all spectra in the line-scan profile across several SrO interfaces. The details of this subtraction are shown in Supplementary Figure 10b.

In order to quantify the hole and Sr concentration for each (La,Sr)O - CuO_2 - (La,Sr)O constituting block, we analyzed the EELS intensity profiles (O-K edge pre-peak and Sr-$L_{2,3}$ edge, respectively) resulting by averaging the results for linescans over 5 different superlattice periods (N = 7). By doing this, we obtained the profiles shown in Supplementary Figure 11 (light blue and orange for Sr and holes, respectively), in which the area of each constituting block has been highlighted (we considered the position of the nominal SrO layer as corresponding to the maximum of the Sr-intensity profile). The difference between the two profiles is clear: symmetric for holes, highly asymmetric for Sr. In order to quantify the Sr and holes

concentration, an average value for each region (dark blue and red points in Supplementary Figure 10) has been defined as the weighted mean of the intensity data referring to the region. The resulting values have been normalized (imposing the sum of the intensities to be equal to 1) in order to satisfy the nominal Sr content and the electroneutrality condition. The outcome is the Sr and holes number per formula unit as shown in Figure 3f and 7.

Zn-tomography

Suppression of T_c upon Cu substitution by Zn in high-T_c superconducting cuprates has been reported several times[10-12]. Zn-doping has been successfully implemented in recent times with the aim of suppressing T_c of single CuO_2 planes in epitaxial thin films of lanthanum cuprate[13] (Zn-tomography).
In order to verify the feasibility of such a method in our case, we compared the electrical properties of ultra-thin (2 u.c.), optimally doped, $La_{1.84}Sr_{0.16}CuO_4$ (exhibiting reduced $T_c \approx 20$ K[14]) and $La_{1.84}Sr_{0.16}Cu_{0.97}Zn_{0.03}O_4$ (Supplementary Figure 12). The transport data confirm the effectiveness of the technique. By reducing the thickness to 2 u.c., we could minimize possible extrinsic effects on T_c due to formation of secondary phases such as ZnO. In Supplementary Figure 13, data from a representative set of samples having the following structure:

$$[8 \text{ x } (\text{LaO-LaO-CuO}_2) + \text{SrO-LaO-CuO}_2 + 8 \text{ x } (\text{LaO-LaO-CuO}_2)]$$

in which different CuO_2 planes have been doped by Zn (3%), are shown. In particular, Supplementary Figure 13a refers to the study of the downward side of the interface (the reference black curve represents the residual resistivity of a sample in which the T_c at the upward side has been suppressed by Zn-doping all the “positive” P planes): one can clearly observe the strong effect of Zn in the suppression of the critical temperature if Zn is put in plane P = -2. In Supplementary Figure 13b, data for the upward side are reported (please note that in this case, P =-2 plane is doped by Zn for all the samples in order to suppress the T_c of the downward side), exhibiting little effect of Zn-doping on the electrical properties. In this case, the scatter in the data can be attributed to differences in sample preparation.

Supplementary References

1 Maier, J. & Pfundtner, G. Defect chemistry of the high-T_c superconductors. *Adv. Mater.* **3**, 292-297, (1991).
2 Maier, J *Physical Chemistry of Ionic Materials* Ch. 6 (Wiley, Chichester, 2004).
3 Opila, E. J., Tuller, H. L., Wuensch, B. J. & Maier, J. Oxygen tracer diffusion in $La_{2-x}Sr_xCuO_{4-y}$ single crystals. *J. Am. Ceram. Soc.* **76**, 2363-2369, (1993).
4 Gozar, A. *et al.* High-temperature interface superconductivity between metallic and insulating copper oxides. *Nature* **455**, 782-785, (2008).
5 Bozovic, I. & Eckstein, J. N. Analysis of growing films of complex oxides by RHEED. *Mrs Bulletin* **20**, 32-38, (1995).
6 Wu, J. et al. Anomalous independence of interface superconductivity from carrier density. *Nat. Mater.* **12**, 877-881, (2013).
7 Parratt, L. G. surface studies of solids by total reflection of x-rays. *Phys. Rev.* **95**, 359-369, (1954).
8 Romberg, H., Alexander, M., Nücker, N., Adelmann, P. & Fink, J. Electronic structure of the system $La_{2-x}Sr_xCuO_{4+\delta}$. *Phys. Rev. B* **42**, 8768-8771, (1990).
9 Chen, C.T. et al. Electronic states in $La_{2-x}Sr_xCuO_{4+\delta}$ probed by soft-x-ray absorption. *Phys. Rev. Lett.* **66**, 104 (1991).

10 Cieplak, M. Z. et al., Metal insulator transition in $La_{1.85}Sr_{0.15}CuO_4$ with various substitutions for Cu. *Phys. Rev. B* **46**, 5536-47, (1992).
11 Nakano, T. et al., Magnetic properties and electronic conduction of superconducting $La_{2-x}Sr_xCuO_4$. *Phys. Rev. B* **49**, 16000-8 (1994).
12 Zagoulaev, S., Monod, P. & Jegoudez, J. Magnetic and transport properties of Zn-doped $YBa_2Cu_3O_7$ in the normal state. *Phys. Rev. B* **52**, 10474-87 (1995).
13 Logvenov, G., Gozar, A. & Bozovic, I. High-temperature superconductivity in a single copper-oxygen plane. *Science* **326**, 699-702, (2009).
14 Sato, H., Yamamoto, H. & Naito, M. Growth of (001) $La_{1.85}Sr_{0.15}CuO_4$ ultrathin films without buffer layers. *Physica C* **274,** 227-231 (1997).
